# Simultaneous Energy Storage and Seawater Desalination using Rechargeable Seawater Battery: Feasibility and Future Directions

**DOI:** 10.1002/advs.202101289

**Published:** 2021-07-28

**Authors:** Moon Son, Sanghun Park, Namhyeok Kim, Anne Therese Angeles, Youngsik Kim, Kyung Hwa Cho

**Affiliations:** ^1^ School of Urban and Environmental Engineering Ulsan National Institute of Science and Technology (UNIST) UNIST‐gil 50 Ulsan 44919 Republic of Korea; ^2^ School of Energy & Chemical Engineering Ulsan National Institute of Science and technology (UNIST) UNIST‐gil 50 Ulsan 44919 Republic of Korea

**Keywords:** cost analysis, desalination, energy storage systems, seawater batteries

## Abstract

Rechargeable seawater battery (SWB) is a unique energy storage system that can directly transform seawater into renewable energy. Placing a desalination compartment between SWB anode and cathode (denoted as seawater battery desalination; SWB‐D) enables seawater desalination while charging SWB. Since seawater desalination is a mature technology, primarily occupied by membrane‐based processes such as reverse osmosis (RO), the energy cost has to be considered for alternative desalination technologies. So far, the feasibility of the SWB‐D system based on the unit cost per desalinated water ($ m^−3^) has been insufficiently discussed. Therefore, this perspective aims to provide this information and offer future research directions based on the detailed cost analysis. Based on the calculations, the current SWB‐D system is expected to have an equipment cost of ≈1.02 $ m^−3^ (lower than 0.60–1.20 $ m^−3^ of RO), when 96% of the energy is recovered and stable performance for 1000 cycles is achieved. The anion exchange membrane (AEM) and separator contributes greatly to the material cost occupying 50% and 41% of the total cost, respectively. Therefore, future studies focusing on creating low cost AEMs and separators will pave the way for the large‐scale application of SWB‐D.

## Graphical abstract

Seawater battery desalination (SWB‐D) is a multifunctional system capable of storing energy and removing salt from water simultaneously. This perspective lays out a detailed cost analysis of the system, and provides an insight as to where SWB‐D currently stands in terms of its feasibility and what areas need to be improved to realize large scale applications.

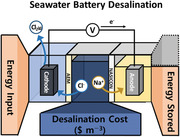

## 1 Introduction

There is great interest in securing alternative water resources at an affordable price, consequently accelerated by the imbalance in accessible water due to climate change.^[^ ^1^ ^]^ In areas adjacent to seawater, freshwater is partially supplied through seawater desalination.^[^ ^2^, ^3^, ^4^ ^]^ However, most conventional processes, which utilize either high temperature (evaporation; >8 kWh m^−3^) or high applied pressure (membrane separation; >3 kWh m^−3^), are known to be energy intensive.^[^ ^3^, ^4^ ^]^ Fortunately, a new concept introduced in the year 2018 has the potential in addressing high desalination energy requirements,^[^ ^5^ ^]^ causing excitement in the water and energy societies. Seawater battery desalination (SWB‐D) uses rechargeable seawater battery (SWB) to save energy used during seawater desalination. It is a multi‐functional process as it simultaneously stores electricity induced by sodium ion movement while removing salt ions from seawater.^[^ ^5^, ^6^, ^7^, ^8^ ^]^ So far, the cost for unit desalinated water from SWB‐D remains unexplored. This is primarily due to the lack of information from previous literatures regarding the energy prices of stand‐alone SWBs. There has been a study that compared the energy price of an entire SWB unit with other energy storage systems (ESS) in a plant‐scale,^[^ ^9^ ^]^ but the price of a unit cell or the detailed energy price divided by cell compartment has not been reported yet. Therefore, this perspective discusses the feasibility of SWB‐D system based on the unit cost per desalinated water ($ m^−3^), which was calculated by the amount of produced water divided by the unit price of SWB‐D. In addition, the future direction for seawater desalination research is also discussed. To accomplish these goals, the fundamental working mechanism of SWB will be first discussed as it works as an anode in SWB‐D system.

## 2 Feasibility of Seawater Battery Desalination System

### 2.1 Seawater Battery: What It Is?

**Figure 1:**
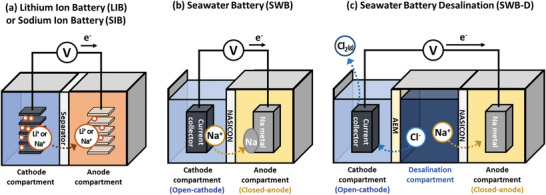
Comparison between a) lithium ion battery (LIB) or sodium ion battery (SIB), b) seawater battery (SWB), and c) simplified seawater battery desalination systems (SWB‐D) upon charging. For SWB‐D system, sodium ions are solidified on the anode and chloride ions migrate to the cathode compartment to maintain charge neutrality while partial energy used during desalination is stored in the SWB anode. Unlike LIB or SIB, SWB, and SWB‐D have an open‐cathode compartment. In addition, sodium super‐conducting separator (NASICON) is used for SWB and SWB‐D, whereas a separator is used for LIB or SIB.

This perspective aims to provide comprehensive understandings and future directions of a new concept of simultaneous energy storage and seawater desalination using SWB. To understand this concept, the working principles of SWB must be discussed first. Unlike lithium ion batteries (LIBs), SWB utilizes sodium ions instead of lithium ions as charge carriers (**Figure** **1**a,b).^[^ ^10^, ^11^ ^]^ SWB is composed of three parts: 1) Open‐cathode compartment for seawater exposure, 2) sodium super‐conducting separator (ceramic based Na_3_Zr_2_Si_2_PO_12_; NASICON), and 3) closed‐anode compartment.^[^ ^11^ ^]^ Water on Earth is 97.5% seawater wherein 30.5% of its total salt is sodium.^[^ ^12^ ^]^ Thus, when SWB is immersed into the seawater, the free and abundant sodium ions in the catholyte can migrate into the anode compartment during charging, storing it as sodium metal. Then, discharging converts the sodium metal back to sodium ions, releasing it into the seawater. Throughout this perspective, we assume that sodium metal anode is used because it is one of the most widely used anodes in SWB research. The continued research during the few past years reveals that SWB is rechargeable (85% energy efficiency over 300 cycles) similar to other ESS technologies.^[^ ^12^, ^13^ ^]^ To simplify our discussion, we are going to discuss about this rechargeable SWB (invented in the year 2014),^[^ ^14^ ^]^ otherwise noticed, although there were non‐rechargeable SWBs (or primary SWBs), which was developed in the year 1943.^[^ ^12^ ^]^

It is worth noting here that SWB is different from sodium ion battery (SIB)^[^ ^15^ ^]^ or desalination battery (DB).^[^ ^16^, ^17^, ^18^ ^]^ SIBs have almost the same structure as LIBs but only use sodium ions and accordingly tuned materials.^[^ ^15^ ^]^ DBs use sodium‐intercalating electrodes (cathode and anode) and a salt solution flowing along the surface of the electrodes.^[^ ^16^, ^17^, ^18^ ^]^ The water flowing channel in the DB is often divided by ion‐exchange membranes in between two electrodes, and redox solution is used as the catholyte and the anolyte. Unlike SIB and DB, SWB system has a unique structure of an open‐cathode compartment with a closed‐anode compartment. Thus, SWB can be operated by immersing it directly into the seawater without using a redox chemical and its associated chambers. In addition, the use of sodium metal anode in SWB enabled a higher capacity compared to DB with sodium intercalation material as anode (≈300 mAh g^−1^ for sodium metal^[^ ^19^ ^]^ and ≈35 mAh g^−1^ for Na_2−x_Mn_5_O_10_ electrode^[^ ^16^ ^]^). In fact, there is another study of SWB where the configuration is similar to DB since ion intercalation electrodes were used. However, relatively low discharging voltage of <1.2 V was reported in that study,^[^ ^20^ ^]^ which is significantly lower than that of typical output voltage of SWB (>3.0 V).^[^ ^12^ ^]^ The reaction mechanisms for each component will not be discussed in this perspective but are available in previous literatures if further understanding is needed.^[^ ^11^, ^12^ ^]^

### 2.2 Is Seawater Battery Competitive to Lithium‐Ion Battery?

**Figure 2:**
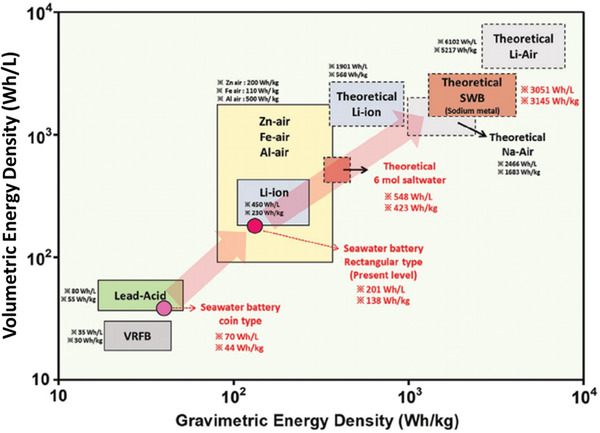
Comparison of the energy density between SWB and other battery systems. Reproduced with permission.^[^ ^21^ ^]^ Copyright 2020, Wiley‐VCH. A typo in the original figure (y‐axis label) was corrected.

To rationalize the use of SWB‐based system in seawater desalination, a brief comparison between SWB and LIB is necessary. For SWB systems, the charging process involves two redox reactions: oxygen evolution reaction on the cathode (*E* ^0^= 0.77 V vs SHE) and sodium solidification on the anode (*E* ^0^= −2.71 V vs SHE).^[^ ^12^ ^]^ Thus, the overall cell reaction in SWB during charging requires 3.48 V (vs Na/Na^+^). Such high voltage of the SWB cell enabled the first coin‐type SWB (SWB_coin_) competitive to lead‐acid battery (70 Wh L^−1^ of SWB_coin_ vs 80 Wh L^−1^ of lead‐acid battery) (**Figure** **2**).^[^ ^21^ ^]^ However, there is still a considerable difference in the energy density between the second generation of rectangular‐type SWB (SWB_Rect_) and conventional LIB (201 Wh L^−1^ of SWB_Rect._ vs 450 Wh L^−1^ of LIB).^[^ ^10^ ^]^ Although the theoretical maximum energy density of SWB is higher than that of LIB (3051 Wh L^−1^ of SWB vs 1901 Wh L^−1^ of LIB),^[^ ^10^ ^]^ more investigations should be carefully carried out to determine the achievable energy density of SWB, and then a reasonable comparison with the corresponding LIB must be made in future research.

To our knowledge, only one discussion was reported about the energy and power costs of SWB compared to other battery systems.^[^ ^9^ ^]^ According to that study, the energy cost for a plant‐size SWB (or full‐size ESS system) is 187 $ kWh^−1^. Although the energy cost is highly dependent on the size of the battery used in the calculation and the life cycle of the total system, the plant‐size calculation of SWB was lower than that of LIB (996–2126 $ kWh^−1^). The low energy cost determined from this study is most likely from the SWB's use of seawater as the catholyte, and the absence of expensive lithium ion. However, the detailed cost analysis for each compartment at a unit cell level and its associated future research directions were not provided in that study. In contrast, a different study comparing SIB and LIB revealed that the cell material cost of SIB (101 $ kWh^−1^ for *β*‐NaMnO_2_) is slightly higher than that of LIB (88 $ kWh^−1^ for LiMn_2_O_4_).^[^ ^22^ ^]^ In terms of cathode material, changing lithium to sodium marginally decreased the total material cost by 3.8%. Conversely, switching from a lithium‐specific anode to a sodium‐specific anode increased the total cost owing to its lower energy density than a lithium‐specific anode. For SIB, the cell material cost was 101 $ kWh^−1^, while the total cost including additional items such as casings, battery management system, and battery thermal management unit was 287 $ kWh^−1^. Based on the above‐mentioned literatures, the significant discrepancy of costs calculated for different scales and materials implies that the compartment cost (or cell material cost) at a unit cell level must be provided for a fair comparison between SWB and LIB as an ESS system. In addition, by using the cost‐related information of SWB for each compartment, we are extending this to SWB‐D systems to determine the unit cost per desalinated water. We are hoping that our calculations and methods can serve as a reference for future SWB studies and their relevant expansion to other technologies.

### 2.3 Seawater Battery Desalination System: Energy Storage System during Desalination

**Figure 3:**
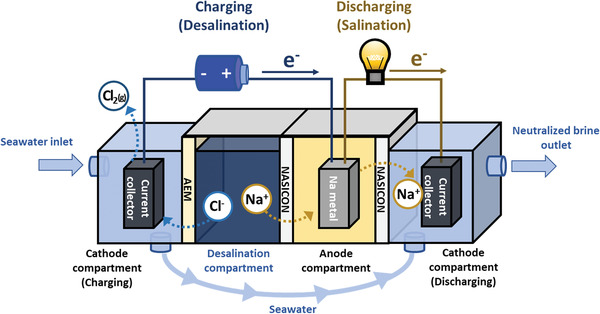
Schematic illustration of the completed SWB‐D system. Upon charging, water is desalinated in the desalination compartment while energy is stored as sodium metal in the anode (blue circuit). During discharging, the water is salinated in the cathode compartment while energy stored in the anode is released (yellow circuit).

The unique sodium adsorption property of SWB led to the development of its expansion systems such as SWB‐D.^[^ ^5^, ^6^, ^7^, ^8^ ^]^ The proposed SWB‐D system can be divided into two parts: Three chambers for charging (desalination) and two chambers for discharging (salination) (**Figure** **3**). Unlike SWB, there is a desalination compartment between the anode and cathode compartments for charging of the system (Figures 1c and 3). The desalination compartment is separated from the cathode compartment by an anion exchange membrane (AEM). Upon charging, sodium ions present in the seawater migrate to the anode compartment and solidify to sodium metal (typically when sodium metal anode is used). Meanwhile, its anion pair (primarily chloride ions) transfers to the cathode compartment through the AEM to maintain charge neutrality in the desalination compartment. The migrated chloride ions can be further removed (partially) through chlorine gas evolution (2 Cl^−^ → Cl_2_(g) + 2 *e* ^−^ *E* ^0^= −1.36 V vs SHE).^[^ ^7^ ^]^ Thus, SWB‐D is a multi‐functional system to store renewable energy during desalination because the partial amount of the energy used during desalination is directly stored into the SWB. The discharging part of the SWB‐D system often shares the anode compartment with the charging part to utilize the energy stored during the charging of the system (Figure 3). Upon discharging of the SWB, the stored energy is released while the dissolved oxygen is reduced (O_2_ + H_2_O + 2*e* ^−^ → HO_2_^−^ + OH^−^ *E* ^0^= 0.21 V vs SHE; O_2_ + 2H_2_O + 4*e* ^−^ → 4OH^−^ *E* ^0^= 0.77 V vs SHE).^[^ ^12^ ^]^ Consequently, sodium ions are migrated from the desalination compartment of the charging part to the cathode compartment of the discharging part (rightest part of Figure 3) during simultaneous operation of the system. Thus, this proposed concept can prevent the salination of the desalinated water during discharging. A proof‐of‐concept of this complete SWB‐D system was proposed in the year 2018^[^ ^5^ ^]^ and was successfully demonstrated in the previous study.^[^ ^7^ ^]^

It is worth mentioning here that nomenclatures of cathode and anode in SWB‐D system are opposite to those in other electrochemical desalination technologies such as capacitive deionization and electrodialysis (ED). For example, sodium ions are attracted to the anode in SWB‐D system, whereas those are adsorbed onto the cathode in other systems. This difference in nomenclature has arisen from the different purposes of the operation. The purpose of battery systems such as SWB‐D is to provide electrons through the outer circuit connected to the cell. In contrast, other electrochemical cells focus more on the electrochemical reactions inside the cell.

### 2.4 Seawater Battery Desalination versus Other Seawater Desalination Technologies

**Figure 4:**
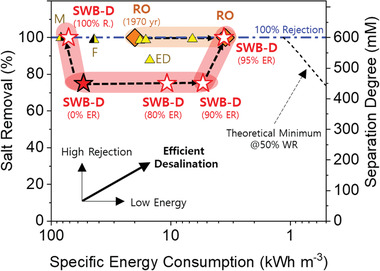
Salt removal (R) as a function of the specific energy consumption for SWB (Red star), reverse osmosis (RO; orange trapezoid), and electrochemical processes for seawater desalination. The three representative electrochemical processes included are electrodialysis (ED; yellow triangle), membrane capacitive deionization (MCDI; M; yellow triangle with black right‐half), and flow‐electrode capacitive deionization (FCDI; F; yellow triangle with black left‐half). The coin‐type SWB (SWB_coin_) was used for the calculation. SWB‐D denotes the desalination system based on SWB_coin_. Note that the theoretical minimum energy for complete seawater desalination was calculated based on 50% of water recovery (WR; black dash line). A seawater concentration of 600 × 10^−3^ m was used for all calculations. ER: energy recovery.

To compare SWB‐D with other seawater desalination technologies, a plot describing salt removal as a function of specific energy consumption (SEC) was created (**Figure** **4**). Since, SWB‐D is an electrochemical method, it was compared with three other electrochemical desalination technologies (membrane capacitive deionization, MCDI; flow‐electrode capacitive deionization, FCDI; and ED). RO was also included in the plot as a reference since it is one of the dominant seawater desalination technologies in the market.

When RO was first commercialized back in the 1970s, its SEC was reported as 20 kWh m^−3^ (orange trapezoid in Figure 4).^[^ ^23^ ^]^ The development of pressure exchangers and highly permeable membranes enabled to reduce this SEC to ≈3.5 kWh m^−3^ starting approximately in the year 2010 (at 50% water recovery).^[^ ^24^, ^25^ ^]^ This number is close to the theoretical minimum energy requirement for seawater desalination (≈1.1 kWh m^−3^ at 50% water recovery; Intersection of black and blue dash lines in Figure 4).^[^ ^26^ ^]^ Thus, RO has been recognized as one of the most promising seawater desalination technologies even though it is still energy intensive compared to wastewater treatment processes for clean water production. In contrast, the two representative capacitive deionization technologies, MCDI and FCDI, have shown relatively high SEC of 83.2 kWh m^−3^ (MCDI)^[^ ^27^ ^]^ and 43.0 kWh m^−3^ (FCDI)^[^ ^28^ ^]^ for seawater desalination. Although MCDI and FCDI have been extensively investigated for brackish water desalination,^[^ ^29^, ^30^, ^31^ ^]^ the application of these processes to seawater desalination has been limited primarily because their SECs proportionally increase with feed salinity, and the salinity of seawater is often ≈35,000 ppm (total dissolved solids; TDS). For ED, several studies have been reported for seawater desalination, which showed SECs of 6.6 kWh m^−3^ (99% salt removal),^[^ ^32^ ^]^ 15 kWh m^−3^ (87% salt removal),^[^ ^33^ ^]^ 16.2 kWh m^−3^ (98% salt removal),^[^ ^34^ ^]^ or 18.1 kWh m^−3^ (98% salt removal).^[^ ^35^ ^]^ These values are distinctly higher than that of RO (3.5 kWh m^−3^ for ≈100% salt removal). Note that the SEC of RO varies from 2.5 to 5 kWh m^−3^ depending on the size of the plant, the number of stages (i.e., single pass), and feed water quality.^[^ ^36^, ^37^, ^38^ ^]^ An SEC of 3.5 kWh m^−3^ was selected as a baseline since this is a common value that often appears in the references.^[^ ^36^, ^37^, ^38^ ^]^

The first reported SEC for SWB‐D is 53.9 kWh m^−3^ (≈75% salt removal) in the year 2020,^[^ ^6^ ^]^ which is similar to MCDI and FCDI. According to literature, the energy consumption of SWB‐D is almost proportional to the amount of salt removed.^[^ ^6^, ^8^ ^]^ Thus, when 100% of salt removal is assumed, the SEC of SWB‐D could proportionally increase to 71.9 kWh m^−3^, not including the increase in salt solution resistance as salt removal reaches 100%. Note that although we ruled out the change of the solution resistance during operation to simplify our discussion, the practical SEC can further increase. However, one of the important aspects of SWB‐D that can distinguish it from other desalination technologies is its energy storing ability during desalination. In practical application, ≈80% of energy recovery (or energy storing) is often reported for SWB.^[^ ^12^ ^]^ This means that 80% of the energy used for charging can be reused for discharging. In this case, the SEC of SWB‐D can decrease to 10.8 kWh m^−3^. When 90% of energy recovery is assumed, which is achievable by reducing the voltage gap during cycling,^[^ ^12^, ^39^ ^]^ the energy consumption further decreases to 5.4 kWh m^−3^. In other literatures, the use of intercalating material as a cathode or redox electrolyte solution as catholyte minimized the voltage gap of SWB.^[^ ^39^, ^40^, ^41^ ^]^ Thus, similar approaches can be adopted for SWB‐D to maximize the energy recovery during desalination. In conclusion, in order for SWB‐D to have similar energy consumption and salt removal rate as RO, it must have an energy recovery rate of 95% (3.6 kWh m^−3^ at 100% salt removal; Open red star in Figure 4) if SWB‐D can be operated solely without any pre‐ and post‐treatment of seawater.

Since SWB‐D system was newly proposed, insufficient information has been reported regarding the influence of feed water quality on the overall system performance. However, from the general engineering point of view, similar pre‐ and post‐treatment of seawater could be required for a full‐scale operation of SWB‐D system. This is because untreated seawater contains several organics/inorganic matters and suspended solid/particles, which can easily decrease the overall performance of the system due to channel‐clogging and contamination (also known as fouling) of the materials. Thus, for the SWB‐D system, additional energy consumption for additional treatment such as intake and pre‐treatment of seawater, and distribution of the produced clean water could be further accounted. Note that these are found to be 0.19 kWh m^−3^ (intake), 0.39 kWh m^−3^ (pre‐treatment), and 0.18 kWh m^−3^ (distribution) for RO system when the energy consumption was divided by the amount of produced water.^[^ ^36^ ^]^ To simplify our discussion, however, those additional energy consumptions were not accounted in this study.

Another factor that should be considered is the salt removal rate of SWB‐D system. Although the maximum salt removal of SWB‐D system remains unexplored primarily due to the voltage threshold of the system, the current design of SWB‐D system can mostly remove Na^+^ and Cl^−^ ions. This is because NASICON allows only Na^+^ ions to pass through and AEM has no specific selectivity toward anions. Thus, the maximum salt removal of the current SWB‐D system is ≈85%, which is the NaCl portion in seawater.^[^ ^12^ ^]^ The remaining ions, primarily divalent sulfate (7.6%), magnesium (3.7%), and calcium (1.2%) should be further treated with other methods. In this regard, post‐treatment using nanofiltration (NF), which is effective to remove divalent ions, can be considered. Thus, an energy consumption of 0.5 kWh m^−3^ for NF post‐treatment was further added (see the Experimental Section/Methods for details). The result showed that the energy consumption of this system (denotes SWB‐D‐NF) is competitive to RO when 96% of energy recovery is achieved (3.4 kWh m^−3^). Thus, the energy recovery ratio of well above 90% should be targeted to render SWB‐D system competitive to RO in terms of energy consumption. Alternatively, energy‐free ion movement, such as diffusion between compartments, particularly between the desalination and cathode compartment, must be considered to reduce the energy used per ion removed. For example, if the ion transportation between the desalination and cathode compartment is promoted via diffusion, the desalination kinetics can be significantly improved without additional energy consumption, thereby lowering the overall SEC used for desalination. Therefore, future studies would need to focus on achieving SECs lower than RO in order for SWB‐D to be competitive in the market of desalination.

### 2.5 Cost Breakdown of Seawater Battery Desalination and Seawater Battery

**Figure 5:**
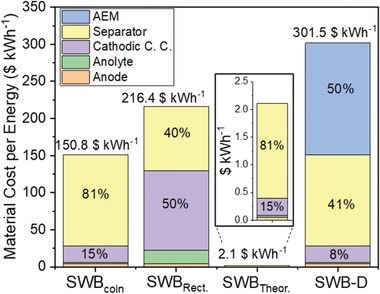
Material cost per energy ($ kWh^−1^) and cost breakdown (%) for SWB_coin_, SWB_Rect._ (rectangular‐type SWB), SWB_Theor._ (theoretical minimum cost), and SWB‐D systems. The cost of SWB_Theor._ and SWB‐D was calculated based on the size and energy efficiency of SWB_coin_. The theoretical energy capacity of 3145 Wh kg^−1^ was applied to calculate SWB_Theor_. cost. The components for the calculation include the anion‐exchange membrane (AEM), separator (NASICON; sodium super‐conducting solid electrolyte), cathodic current collector (cathodic C. C.), anolyte, and anode. Note that the material cost of LIB ranged from ≈88 to ≈200 $ kWh^−1^.^[^ ^22^, ^42^, ^43^ ^]^

Similar with other processes, the price of the system dictates the price of the product. To estimate the price of clean water produced, the system price of SWB at a unit cell level was calculated. The energy normalized material cost (unit of $ kWh^−1^) was calculated based on the assessable retail price (**Figure** **5**) although material cost could be significantly reduced by bulk purchasing of chemicals. Thus, the material cost presented in this section aims to provide a simplified number for future reference, not to feature the exact number for the current energy price of SWB based systems.

The material cost for two different sizes of SWB (SWB_coin_ and SWB_Rect._) was first calculated. For SWB_coin_, the overall material cost is 150.8 $ kWh^−1^ in which the separator (NASICON) occupied 122 $ kWh^−1^ followed by cathodic current collector (cathodic C. C.; 23 $ kWh^−1^), anode (4 $ kWh^−1^), and anolyte (2 $ kWh^−1^). For SWB_Rect._, which has a larger cathodic C.C. than SWB_coin_ (≈2 cm^2^ for SWB_coin_ and ≈68 cm^2^ for SWB_Rect._), the overall material cost is 216.4 $ kWh^−1^. The material cost for the cathodic C. C. (108 $ kWh^−1^) is dominant for SWB_Rect._ followed by the separator (86 $ kWh^−1^), anolyte (18 $ kWh^−1^), and anode (4 $ kWh^−1^). The material cost of LIB was reported in the range of ≈88 to ≈200 $ kWh^−1^.^[^ ^22^, ^42^, ^43^ ^]^ Thus, the material cost of SWBs (150.8 $ kWh^−1^ for SWB_coin_ and 216.4 $ kWh^−1^ for SWB_Rect._) is reasonable or even cheaper compared to LIB for ESS applications. Note that a significantly higher price of >1700 $ kWh^−1^ was reported for LIBs (year 2007, battery pack cost^[^ ^44^ ^]^) in the initial stage of the development. Thus, the SWB cost can be further reduced based on the fact that retail prices were mostly used for SWB calculations presented in this study. For example, when retail prices were considered for cathode in LIB, which is ≈25% of the overall cost, ≈300 $ kWh^−1^ was calculated (see Detailed Information for Calculations section). However, only ≈26 $ kWh^−1^ was reported for LIB cathode when it was fully commercialized.^[^ ^22^ ^]^ Therefore, as SWB matures, it is expected that its current material cost can be reduced significantly. Moreover, if SWB_coin_ reaches its maximum energy density (3145 Wh kg^−1^), the cost of SWB can reach a theoretical minimum of 2.1 $ kWh^−1^ (SWB_Theor_), which makes it very promising for commercialization.

To calculate the material cost of SWB‐D, only the AEM price of ≈150 $ kWh^−1^ was added to SWB_coin_ resulting in an overall material cost of 301.5 $ kWh^−1^. For SWB‐D, the material cost of AEM and separator occupied ≈91% of the overall cost. Without these compartments, it can go down to ≈28 $ kWh^−1^. Thus, the gradual decrease in AEM and separator costs can render SWB‐D more feasible in terms of energy cost. Furthermore, decreasing the cost of cathodic C.C. also needs to be considered. Current SWB‐D research is based on the SWB_coin_ architecture because it is in the early stage of development. But as presented earlier, cathodic C.C. accounted for 50% of the total cost of SWB_Rect_ with a cathodic C.C. 34 times larger than SWB_coin_. Therefore, once SWB‐D is upscaled, cathodic C.C. would have a large impact on the overall cost.

## 3 Future Directions for Practical Applications

The SWB‐D process has been tested only in a steady‐state (or batch) mode so far, so there was not much consideration for limiting factors such as the diffusion of ions and conductivity of influent solution. Thus, in order to fairly compare SWB‐D technology with other seawater desalination technologies, the development of a continuous process such as a flow‐cell must be investigated. When manufacturing a flow‐cell, an architecture design that can maximize the contact area between the SWB and the desalination compartment is required. It is known that the time taken for desalination in SWB‐D is proportional to the amount of current flowing through the system.^[^ ^6^ ^]^ For the current SWB‐D system, it takes >30 h to desalinate (≈75%) ≈3.4 mL of seawater.^[^ ^6^ ^]^ Therefore, if a flow‐cell, which maximizes the contact area between the anode and seawater, is proposed, the time required for desalination can be drastically reduced. In a batch mode system, solution resistance increases as the desalination progresses resulting to a rapid increase in cell voltage. In contrast, a continuous mode system continuously feeds the cell with high concentration influent, which prevents the increase in solution resistance and reduces the rate of increase in cell voltage. In this way, the amount of current applied per volume of seawater can be maximized in a flow‐cell. For this purpose, a rectangular or pouch type developed for SWB system can be directly used for SWB‐D system.^[^ ^21^ ^]^ Since most RO processes have been operated under the cross‐flow mode, the development of a flow‐cell particularly for the desalination compartment could lead to a fair comparison between SWB‐D and RO in future studies.

As reported in the previous literature,^[^ ^6^, ^8^ ^]^ SWB‐D system can be applied to hypersaline water treatment owing to a great ion deposition capacity of the SWB anode. However, the energy recovery efficiency in the actual SWB‐D system remains unknown, whereas it is well known in SWB system, which does not possess a desalination compartment. For SWB‐D, it is common to charge with SWB‐D cells (composed of three compartments) and discharge with SWB cells (composed of two compartments) because a flow‐cell (or continuous flow) for SWB‐D has not yet been developed. In this regard, the energy recovery of 100% was assumed in the hybrid process,^[^ ^6^ ^]^ which is difficult to achieve in the practical operation. Therefore, the charging and discharging performance of SWB‐D has to be further conducted in the same flow cell to confirm the practical energy efficiency of SWB‐D system.

By multiplying SEC (kWh m^−3^) and the material cost ($ kWh^−1^), the water production cost ($ m^−3^) can be calculated, which varies depending on the lifespan (or long‐term efficiency) of the system. For example, the water cost of SWB‐D‐NF could be 1.02 $ m^−3^ to desalinate seawater (0.6 m NaCl at 100% removal of salts), which is competitive to RO (0.60–1.20 $ m^−3[^ ^37^ ^]^), when the cycling performance is maintained for 1000 cycles (at 96% energy recovery). Conversely, increasing the power density of the SWB‐D can be an alternative approach that does not require energy efficiency considerations over massive cycles of operation. Using relatively inexpensive membranes instead of AEM could be an alternative approach to reduce water production cost because AEM cost is 50% of the total material cost in SWB‐D. The use of inexpensive membranes might make it possible to divide the desalination compartment of SWB‐D into several sub‐compartments. This alternative approach could reduce energy consumption per ion removal because more ions can be transported through ion exchange membranes with the same energy consumption. As proven by ED research,^[^ ^45^ ^]^ this approach can achieve low energy consumption for ion separation. Using an alternative membrane to AEM, which could facilitate ion diffusion from desalination to cathode compartment could also be a promising option to improve the desalination kinetics of SWB‐D system. Since NASICON has been known as the resistance determining component in SWB‐D system,^[^ ^8^ ^]^ developing a highly conductive NASICON could lead to lower ohmic and diffusion resistance, thereby using a higher applied current would be possible. Note that the NASICON used in current SWB‐D studies has a chemical composition of Na_3_Zr_2_Si_2_PO_12_.^[^ ^8^ ^]^ For example, highly conductive NASICONs such as Na_3.1_Si_2.3_Zr_1.55_P_0.7_O_11_^[^ ^46^ ^]^ or Na_3.4_Zr_2_Si_2.4_P_0.6_O_12_^[^ ^47^ ^]^ could be suitable candidates (i.e., the resistance of ≈10^−4^ S cm^−1^ for Na_3_Zr_2_Si_2_PO_12_ and 5 × 10^−3^ S cm^−1^ for Na_3.4_Zr_2_Si_2.4_P_0.6_O_12_^[^ ^47^ ^]^) for SWB‐D application. Another approach that can be considered to improve the desalination kinetics is the use of redox chemistry in cathode or catholyte as shown in the previous SWB study.^[^ ^48^ ^]^ In this case, the sluggish kinetics of oxygen evolution and reduction reactions can be replaced with relatively fast redox reactions, thereby improving the desalination kinetics of SWB‐D systems.

Designing a large‐scale SWB‐D cell with a minimized cost of cathodic C. C. would also be beneficial as the cathodic C. C. cost is ≈50% in SWB_Rect._. The anode compartment design will have a marginal impact on the overall material cost; however, it can render the system more compact, thereby minimizing the capital cost of any SWB based system including SWB‐D system.

Based on the aforementioned discussions, important concluding remarks can be made as follows: The material costs of AEM (50%) and separator (41%) are current hurdles for large‐scale application of SWB‐D. Energy recovery and cycling efficiency play crucial roles in determining the feasibility of SWB‐D compared to other desalination technologies such as RO. When energy recovery of ≈96% and stable performance for 1000 cycles are achieved, an equipment cost of ≈1.02 $ m^−3^ can be expected, which is similar to RO (0.60–1.20 $ m^−3^). Flow‐cell (or continuous system) development for SWB‐D is urgently required for comprehensive comparisons. Continuous flow could facilitate ion diffusion across the AEM, which could remove more ions without additional energy input. For SWB‐D to compete with other seawater desalination processes, particularly RO, in addition to energy aspects, desalination kinetics must be significantly improved. For future studies, a more realistic cost analysis for large‐scale SWB‐D system can be done when material processing and casing costs are included.

## 4 Experimental Section

### Specific Energy Consumption

Whenever seawater is used for calculation, the salt concentration was assumed as 0.6 m (≈35 g L^−1^ NaCl).^[^ ^26^ ^]^ The salt removal of RO was assumed 100% because ≈99.7% of salt removal has been often reported.^[^ ^49^, ^50^, ^51^ ^]^ For the calculation of the SEC of SWB‐D, it was assumed that salt removal is proportional to energy consumption.^[^ ^6^ ^]^ An energy consumption of 0.5 kWh m^−3^ was assumed for NF in SWB‐D‐NF at a feed concentration of 5200 ppm (when SWB‐D removed 85% of salt from 35 000 ppm TDS). Moreover, a linear relationship between energy consumption and feed TDS in NF was used.^[^ ^52^ ^]^ Note that the numbers used are an approximation and more research is needed to verify the maximum salt removal rate of SWB‐D‐NF using real seawater. This number Theoretical minimum energy required for seawater desalination was calculated by Gibbs free energy of separation at 50% of water recovery.^[^ ^26^, ^53^ ^]^

### Detailed Information for Cost Calculations

The components used in calculations for SWB_coin_ were 0.8 g (NASICON), ≈2 cm^2^ (cathodic C. C.; carbon felt), 15 µL (anolyte; 1 m Biphenyl in diethylene glycol dimethyl ether), and ≈1.5 cm^2^ (anode; stainless steel mesh). For NASICON price, a mass‐based element ratio was applied to each chemical needed to synthesize. Retail prices were used for the chemicals used during NASICON synthesis. The dimensions used in calculations for SWB_Rect._ were 23.6 g (NASICON), 396 cm^2^ (cathodic C. C.; carbon felt), 6 mL (anolyte; 1 m Biphenyl in diethylene glycol dimethyl ether), and ≈68.3 cm^2^ (anode; stainless steel mesh). For the material cost of SWB‐D, which was calculated based on SWB_coin_, an AEM area of ≈2 cm^2^ was considered. The AEM price was calculated based on the minimum unit price (200 $ m^−1^) multiplied by the area used (≈2 cm^2^).^[^ ^54^ ^]^

For LIB calculations using retail prices, a coin cell (2325 coin cell; diameter ≈0.905 in; depth ≈0.098 in) with similar dimensions to SWBcoin was used. Accessible retail prices were used for the chemicals needed for the synthesis of each cell component. The cathode consists of LiNi_0.6_Mn_0.2_Co_0.2_O_2_ (also known as NMC622), carbon black, polyvinylidene fluoride, and *n*‐methyl‐2‐pyrrolidone (NMP). Aluminum foil was used as the cathodic C. C. and the electrolyte was a mixture of LiPF_6_, ethylene carbonate, and diethyl carbonate. Polyethylene membrane was used as the separator. The anode consisted of graphite, carboxymethyl cellulose and styrene‐butadiene rubber, carbon black, and NMP.

## Conflict of Interest

The authors declare no conflict of interest.
